# Acute glycemic variability and mortality of patients with acute stroke: a meta-analysis

**DOI:** 10.1186/s13098-022-00826-9

**Published:** 2022-05-10

**Authors:** Jinbo Lin, Chunsheng Cai, Yituan Xie, Li Yi

**Affiliations:** 1 Department of Neurology, Huizhou First Hospital, Huizhou, 516000 China; 2 Department of Neurology, Peking University Shenzhen Hospital, 1120 Lianhua Road, Shenzhen, 518000 China

**Keywords:** Glycemic variability, Acute stroke, Mortality, Cohort studies, Meta-analysis

## Abstract

**Background:**

Increased glucose fluctuation has been related to poor prognosis in patients with critical illnesses, while its prognostic role in patients with acute stroke remains unknown. The meta-analysis aimed to evaluate the association between the acute glycemic variation (GV) and mortality risk in patients with acute stroke.

**Methods:**

Cohort studies were obtained by searching Medline, Web of Science, Embase, Wanfang and CNKI databases. A random-effect model which incorporates the intra-study heterogeneity was chosen to pool the results.

**Results:**

Ten cohort studies with 1433 patients were included, and 280 (19.5%) of them died within 90 days of disease onset. Results of the meta-analyses showed that a higher acute GV was associated with an increased risk of early mortality in patients with acute stroke, as indicated by GV measured with the coefficient of variation of blood glucose (CVBG, odds ratio [OR]: 2.24, 95% CI 1.40 to 3.58, p < 0.001, I^2^ = 73%), the standard deviation of blood glucose (SDBG, OR: 2.31, 95% CI 1.70 to 3.13, p < 0.001, I^2^ = 50%), and the mean amplitude of glycemic excursion (OR: 3.57, 95% CI 1.44 to 8.85, p = 0.006, I^2^ = 23%). For acute GV measured with CVBG and SDBG, subgroup analyses showed consistent results in patients with acute ischemic and hemorrhagic stroke, and for studies reporting 28-day and 90-day all-cause mortality (p for subgroup analysis all > 0.05).

**Conclusions:**

Higher acute GV may be an independent risk factor of early mortality in patients with acute stroke.

## Introduction

Stroke is a category of acute cerebral vascular disease that significantly threatens the global population’s health [1, 2]. In China, it is estimated that currently, over 2 million new cases of stroke are diagnosed annually [3]. According to the data at the beginning of the twenty-first century approximately 1.1 million inhabitants of Europe suffered a stroke each year [4]. Pathophysiologically, stroke is defined as an abrupt neurological outburst caused by impaired perfusion through the blood vessels to the brain [5]. Despite the continuous efforts in the prevention and treatment of the disease, particularly for the various reperfusion therapies [5], stroke remains one of the leading causes of morbidity and mortality for people worldwide, particularly in developing countries [6, 7]. Accumulating evidence suggests that dysglycemia, including stress-induced hyperglycemia [8], persistent hyperglycemia [9], as well as hypoglycemia [10], are all associated with poor prognosis in patients with acute stroke, which suggests the possible role of glucose fluctuation as a predictor of poor outcomes in patients with acute stroke [11]. Recently, acute glycemic variability (GV), which reflects the extent of glucose fluctuation within days, has been related to poor prognosis in patients with critical illnesses [12–14]. Although no consensus has been reached regarding the optimal measuring method and cutoff of GV in an acute setting, some parameters have been well applied in previous researches, including the coefficient of variation of blood glucose (CVBG), the standard deviation of blood glucose (SDBG), and the mean amplitude of glycemic excursion (MAGE) [15–19]. Using these parameters, some pilot studies have been performed to evaluate the association between acute GV and mortality risk in patients with acute stroke [20–29]. However, the results of these studies were not consistent and the prognostic role of acute GV in patients with acute stroke remains unknown. Therefore, we performed a meta-analysis to systematically investigate the possible predictive role of acute GV for mortality risk in patients with acute stroke.

## Methods

The Preferred Reporting Items for Systematic Reviews and Meta-Analyses (PRISMA 2020) [30, 31] guideline and Cochrane’s Handbook for Systematic Review and Meta-analysis [32] were followed in this study.

### Study search

Studies were obtained by a search of Medline, Web of Science, Embase, Wanfang and CNKI electronic databases using strategy based on the combined keywords: (1) “glycemic” OR “glyceamic” OR “glucose”; (2) “variability” OR “variation” OR “fluctuation”; and (3) “stroke” OR “transient ischemic stroke” OR “TIA” OR “cerebral infarction” OR “cerebrovascular infarction” OR “intracranial hemorrhage” OR “intracerebral hemorrhage”. Only clinical studies were included. No restriction was applied to the publication language. We also screened the citation lists of the related original and review papers in a manual manner as a complementation. The last literature search was conducted on October 15, 2021.

### Study inclusion and exclusion criteria

The PICOS criteria were used for study inclusion.

1. P (Participants): Patients with new-onset acute stroke, including ischemic stroke (AIS) and/or hemorrhagic stroke (AHS);
2. I (Intervention/exposure): Patients with higher acute GV at admission;
3. C (Control/comparator): Patients with lower acute GV at admission;
4. O (Outcome): Incidence of all-cause mortality during follow-up;
5. S (Study design): Cohort studies, including prospective or retrospective cohorts;

Measuring of acute GV was consistent with methods used among the included studies with at least one of the following parameters, including CVBG, SDBG, and MAGE. The incidence of all-cause mortality during follow-up was compared between patients with the highest versus the lowest category of GV, and only studies with multivariate analyses were included.

Reviews, preclinical studies, studies including non-stroke patients, studies that did not measure acute GV, or studies that did not report the outcome of interest were excluded. We did not consider unpublished data because these materials may not be reliable because they were not peer-reviewed.

### Extraction of data and evaluation of study quality

Two independent authors conducted database search, data collection, and assessment of study quality separately. In case of disagreement, it was resolved by discussing with the corresponding author. The data collected were: (1) general study information and study design; (2) patient characteristics, including diagnosis, age, sex, and diabetic status; (3) parameters for the measuring of GV, cutoffs, and duration of GV measurements; (4) follow-up durations and number of patients died during follow-up; and (5) variables adjusted. The Newcastle–Ottawa Scale (NOS) [33] was used for assessing the quality of the studies.

### Statistical methods

The association between acute GV at admission and mortality risk during follow-up in patients with acute stroke was presented as odds ratio (OR) and its 95% confidence intervals (CIs). Logarithmical transformation of OR data and stand error (SE) extracted from each study were performed to achieve a normalized distribution [34]. The Cochrane’s Q test was performed to evaluate the extent of between-study heterogeneity, and the I^2^ statistic was estimated as previously described [34, 35]. An I^2^ > 50% reflected significant heterogeneity. A random-effect model was applied to pool the results after incorporating possible between-study heterogeneity [32]. If possible, subgroup analyses were performed to evaluate the possible influences of study characteristics on the outcome, such as the type of stroke and the follow-up durations. Funnel plots were constructed and visual inspection of their symmetry was performed to assume the possible existence of publication bias [36]. Egger’s regression test [36] was also performed to test possible publication bias. We used RevMan (Version 5.1; Cochrane Collaboration, Oxford, UK) and STATA software for the statistical analyses and a p < 0.05 suggests statistical significance.

## Results

### Study identification

**Fig. 1:**
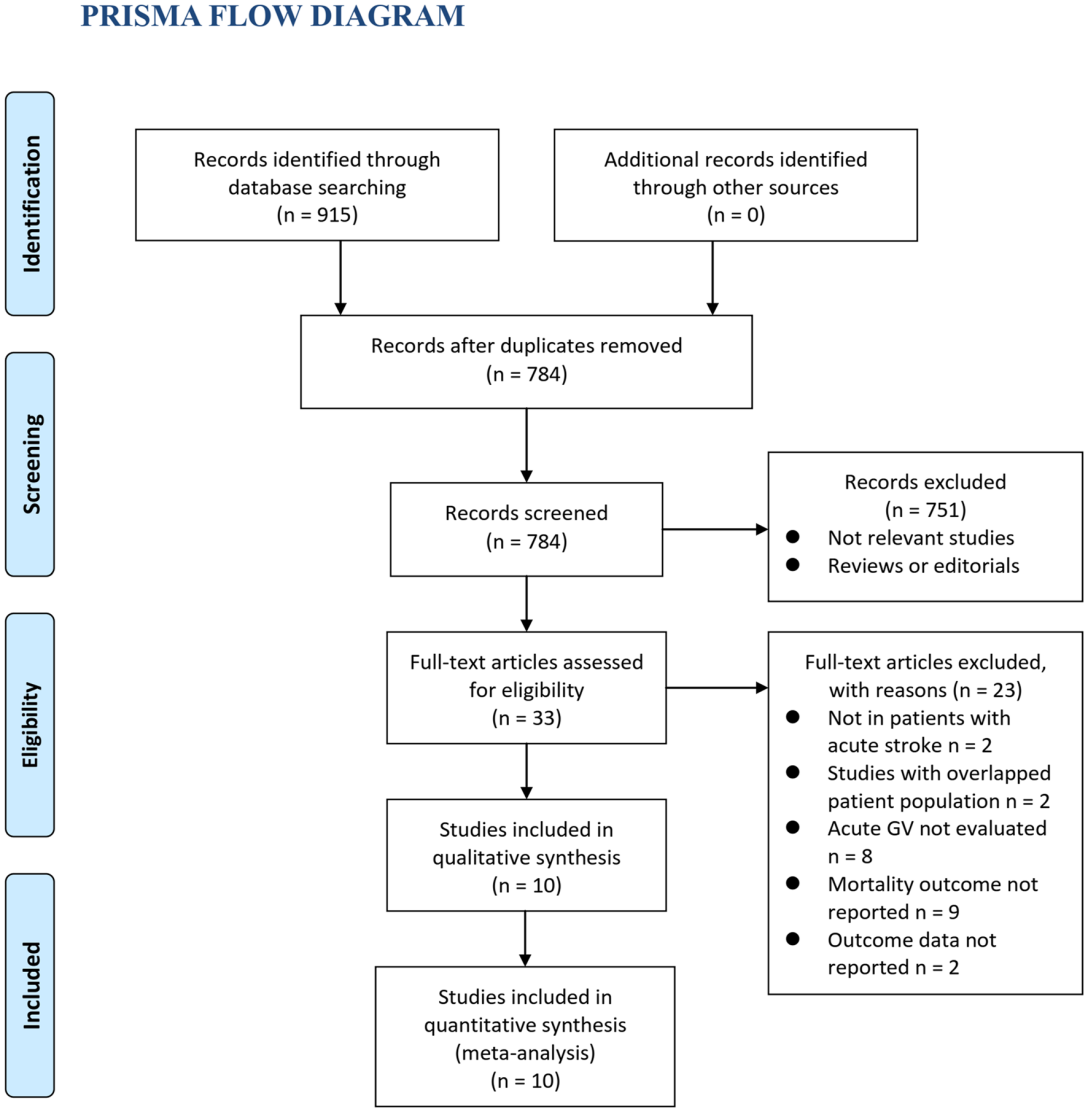
Flowchart of the database search and study identification

As shown in Fig. 1, 784 articles were retrieved after the search of electronic databases after removing duplications. Subsequently, 751 were further excluded due to lacking of relevance. The remaining 33 studies were screened with full text, and 23 were further removed for the reasons in Fig. 1. Finally, ten cohort studies [20–29] were available for the meta-analysis.

### Characteristics of the included studies

Overall, ten retrospective cohort studies with 1433 patients with acute stroke were included [20–29]. Five of them were published in English [21, 23, 24, 27, 29], and the other five were published in Chinese [20, 22, 25, 26, 28]. The characteristics of the included cohort studies were shown in Table 1. These studies were performed in China, Korea, and Spain, and published between 2013 and 2020. Patients with AIS were included in five studies [20, 21, 24, 25, 29], those with AHS were included in two studies [22, 28], while the remaining three studies included both AIS and AHS patients [23, 26, 27]. The mean ages of the included patients varied between 58 and 72 years, and the proportions of men ranged between 47 and 67%. Acute GV was evaluated at admission with CVBG, SDBG, or MAGE, and categorized with different cutoffs. The duration for acute GV measuring varied between 24 and 72 h. The follow-up durations were 28 days for four studies [20, 22, 25, 28], and 90 days for the other six studies [21, 23, 24, 26, 27, 29]. Overall, 280 (19. 5%) patients died during follow-up. Variables including age, comorbidities, baseline National Institute of Health stroke scale (NIHSS), and Acute Physiology and Chronic Health Evaluation II (APACHE-II) Scale etc. were adjusted to a different degree among the included studies. The NOS of the included studies were eight to nine studies, suggesting good quality (Table 2).

**Table 1:** Characteristics of the included cohort studies

Study	Design	Country	Diagnosis	Sample size	Mean age (years)	Male (%)	DM (%)	GV measurements and cutoff	Duration for GV measurements (h)	Follow-up duration (days)	No. of patients died	Variables adjusted
Chen 2013	RC	China	AIS	72	61.7	47.2	0	CVBG (30%)	72	28	12	Age and NIHSS at admission
Yoo 2014	RC	Korea	AIS	207	70.6	61.4	21.3	SDBG (1.2 mmol/l)	24	90	33	Age, NIHSS at admission, AF, HTN, CAD, smoking and SBP
Guo 2015	RC	China	AHS	90	62.8	55.6	NR	CVBG (50%)	72	28	38	Age, NIHSS at admission, APACHE-II Score, and hypoglycemia
Di 2016	RC	China	AIS and AHS	176	60.3	63.6	39.8	CVBG (50%), SDBG (1.2 mmol/l), and MAGE (3.9 mmol/l)	24	90	27	Age, NIHSS at admission, and APACHE-II Score
Wang 2018	RC	China	AIS	111	61.7	58.6	52.3	CVBG (30%) and SDBG (1.3 mmol/l)	24	90	23	Age and APACHE-II Score
Liu 2019	RC	China	AIS and AHS	162	58.5	54.3	42.6	CVBG (50%) and SDBG (1.3 mmol/l)	72	90	30	Age, NIHSS at admission, and APACHE-II Score
Cui 2019	RC	China	AIS	107	63.5	54.2	57.9	CVBG (30%) and SDBG (1.4 mmol/l)	72	28	35	Age and APACHE-II Score
Gutiérrez 2020	RC	Spain	AIS	213	71.2	60.1	30	SDBG (median)	48	90	16	Age, NIHSS at admission, and comorbidities
Cai 2020	RC	China	AIS and AHS	158	65.9	63.3	39.2	CVBG (50%), SDBG (1.3 mmol/l), and MAGE (median)	24	90	24	Age, NIHSS at admission, APACHE-II Score, and mean BG
Chen 2020	RC	China	AHS	137	60.6	66.4	NR	CVBG (20%) and SDBG (1.4 mmol/l)	24	28	42	Age and APACHE-II Score

AIS, acute ischemic stroke; AHS, acute hemorrhagic stroke; RC, retrospective cohort; DM, diabetes mellitus; GV, glycemic variability; NR, not reported; AF, atrial fibrillation; HTN, hypertension; CAD, coronary artery disease; AF, atrial fibrillation; SBP, systolic blood pressure; BG, blood glucose; CVBG, coefficient of variation of blood glucose; SDBG, standard deviation of blood glucose; MAGE, mean amplitude of glycemic excursion; NIHSS, National Institute of Health stroke scale; APACHE-II, Acute Physiology and Chronic Health Evaluation II

**Table 2:** Details of quality evaluation via the Newcastle–Ottawa Scale

Study	Representativeness of the exposed cohort	Selection of the non-exposed cohort	Ascertainment of exposure	Outcome not present at baseline	Control for age	Control for other confounding factors	Assessment of outcome	Enough long follow-up duration	Adequacy of follow-up of cohorts	Total
Chen 2013	0	1	1	1	1	1	1	1	1	8
Yoo 2014	0	1	1	1	1	1	1	1	1	8
Guo 2015	0	1	1	1	1	1	1	1	1	8
Di 2016	1	1	1	1	1	1	1	1	1	9
Wang 2018	0	1	1	1	1	1	1	1	1	8
Liu 2019	0	1	1	1	1	1	1	1	1	8
Cui 2019	0	1	1	1	1	1	1	1	1	8
Gutierrez 2020	1	1	1	1	1	1	1	1	1	9
Cai 2020	1	1	1	1	1	1	1	1	1	9
Chen 2020	0	1	1	1	1	1	1	1	1	8

### Overall meta-analysis results

**Fig. 2:**
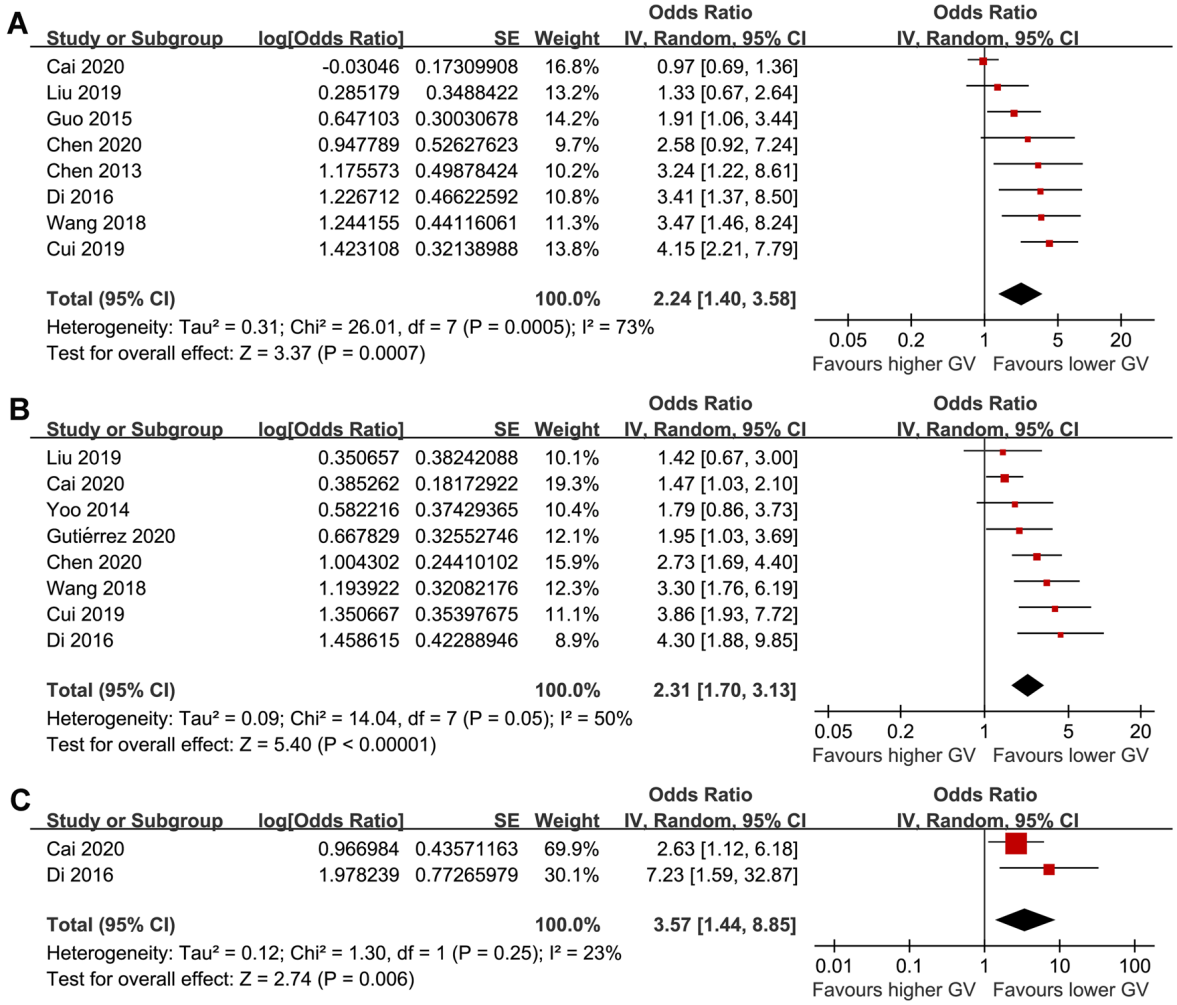
Forest plots for the meta-analysis of the association between acute GV and mortality risk in patients with acute stroke; **A** meta-analysis of GV measured by CVBG; **B** meta-analysis of GV measured by SDBG; and **C** meta-analysis of GV measured by MAGE

A meta-analysis of eight studies [20, 22–28] showed that a higher acute GV measured by CVBG was associated with an increased risk of early mortality in patients with acute stroke (OR: 2.24, 95% CI 1.40 to 3.58, p < 0.001, I^2^ = 73%; Fig. 2A). Besides, pooled results of eight studies [21, 23–29] showed that a higher acute GV measured by SDBG was also associated with an increased risk of early mortality (OR: 2.31, 95% CI 1.70 to 3.13, p < 0.001, I^2^ = 50%; Fig. 2B). Similarly, pooled results of two studies with GV measured by MAGE showed consistent result (OR: 3.57, 95% CI 1.44 to 8.85, p = 0.006, I^2^ = 23%; Fig. 2C).

### Subgroup analyses

**Fig. 3:**
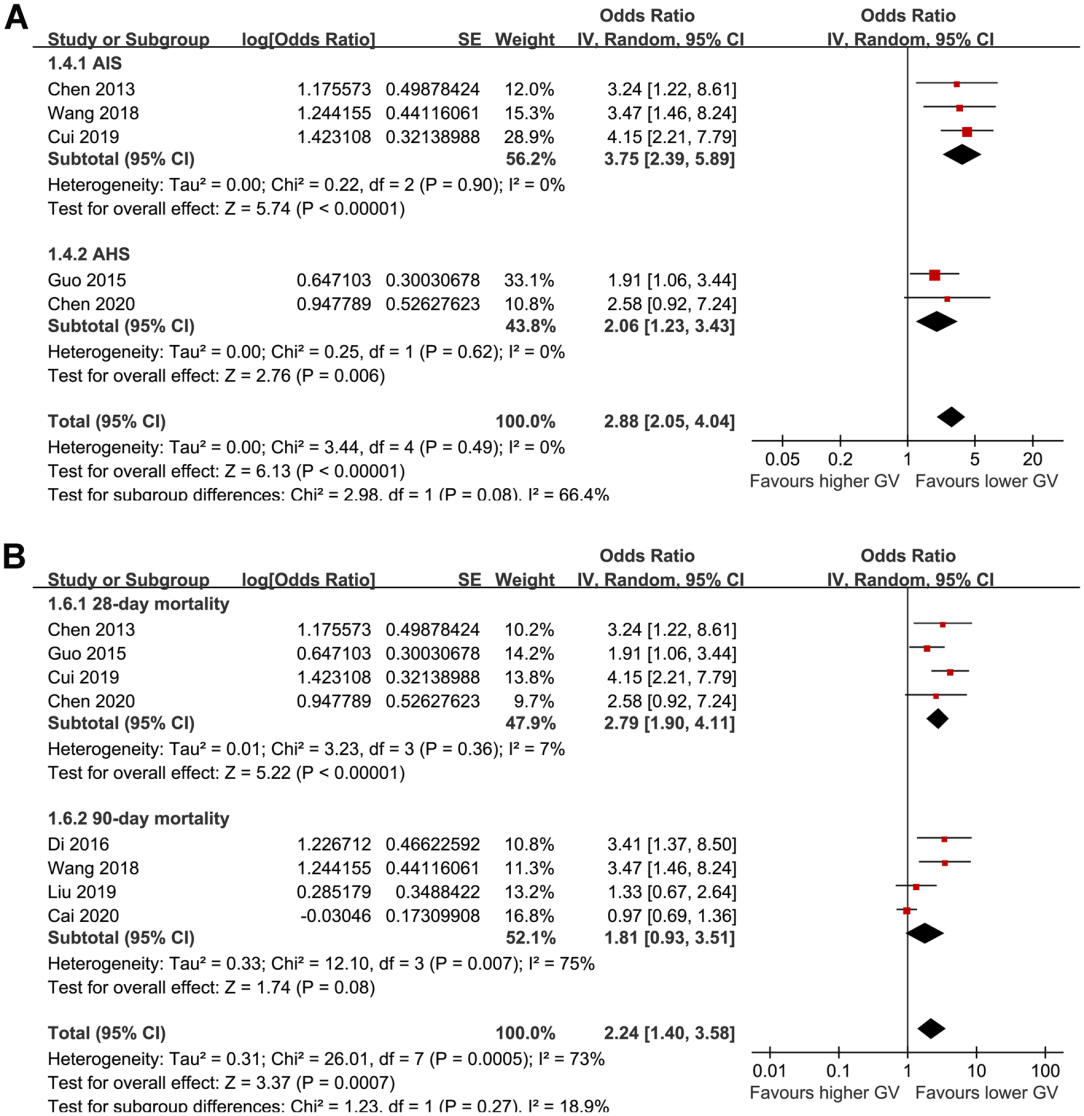
Subgroup analysis for the meta-analysis of CVBG and mortality risk in patients with acute stroke; **A** subgroup analysis according to the type of stroke; and **B** subgroup analysis according to the follow-up duration

For studies of acute GV measured with CVBG, subgroup analysis showed that a higher acute GV was associated with increased mortality risk in patients with AIS (OR: 3.75, 95% CI 2.39 to 5.89, p < 0.001; I^2^ = 0%) and in patients with AHS (OR: 2.06, 95% CI 1.23 to 3.43, p = 0.006; I^2^ = 0%; p for subgroup difference = 0.08; Fig. 3A). Subgroup analysis according to the follow-up duration showed that higher CVBG was associated with an increased risk of 28-day mortality (OR: 2.79, 95% CI 1.90 to 4.11, p < 0.001; I^2^ = 7%), while the association between CVBG and 90-day mortality was not statistically significant (OR: 1.81, 95% CI 0.93 to 3.51, p = 0.08; I^2^ = 75%). However, the difference between subgroup analysis was not statistically significant (p for subgroup difference = 0.27; Fig. 3B).

**Fig. 4:**
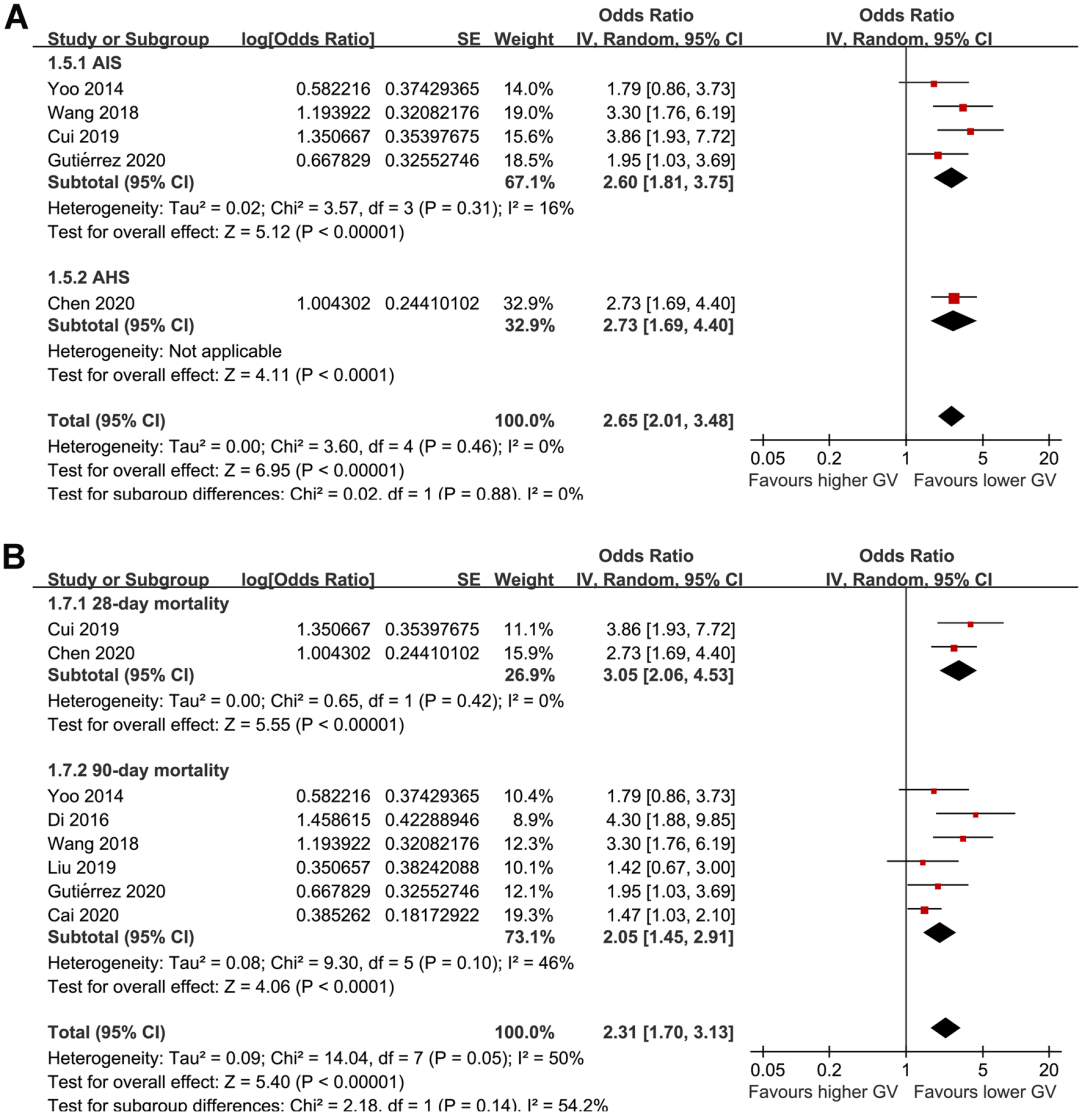
Subgroup analysis for the meta-analysis of SDBG and mortality risk in patients with acute stroke; **A** subgroup analysis according to the type of stroke; and **B** subgroup analysis according to the follow-up duration

For acute GV measured with SDBG, subgroup analyses showed consistent results in patients with AIS (OR: 2.60, 95% CI 1.81 to 3.75, p < 0.001; I^2^ = 16%) and ASH (OR: 2.73, 95% CI 1.69 to 4.40, p < 0.001; p for subgroup difference = 0.88; Fig. 4A), and for studies reporting 28-day (OR: 3.05, 95% CI 2.06 to 4.53, p < 0.001; I^2^ = 0%) and 90-day all-cause mortality (OR: 2.05, 95% CI 1.45 to 2.91, p < 0.001; I^2^ = 46%; p for subgroup difference = 0.14; Fig. 4B).

### Publication bias

**Fig. 5:**
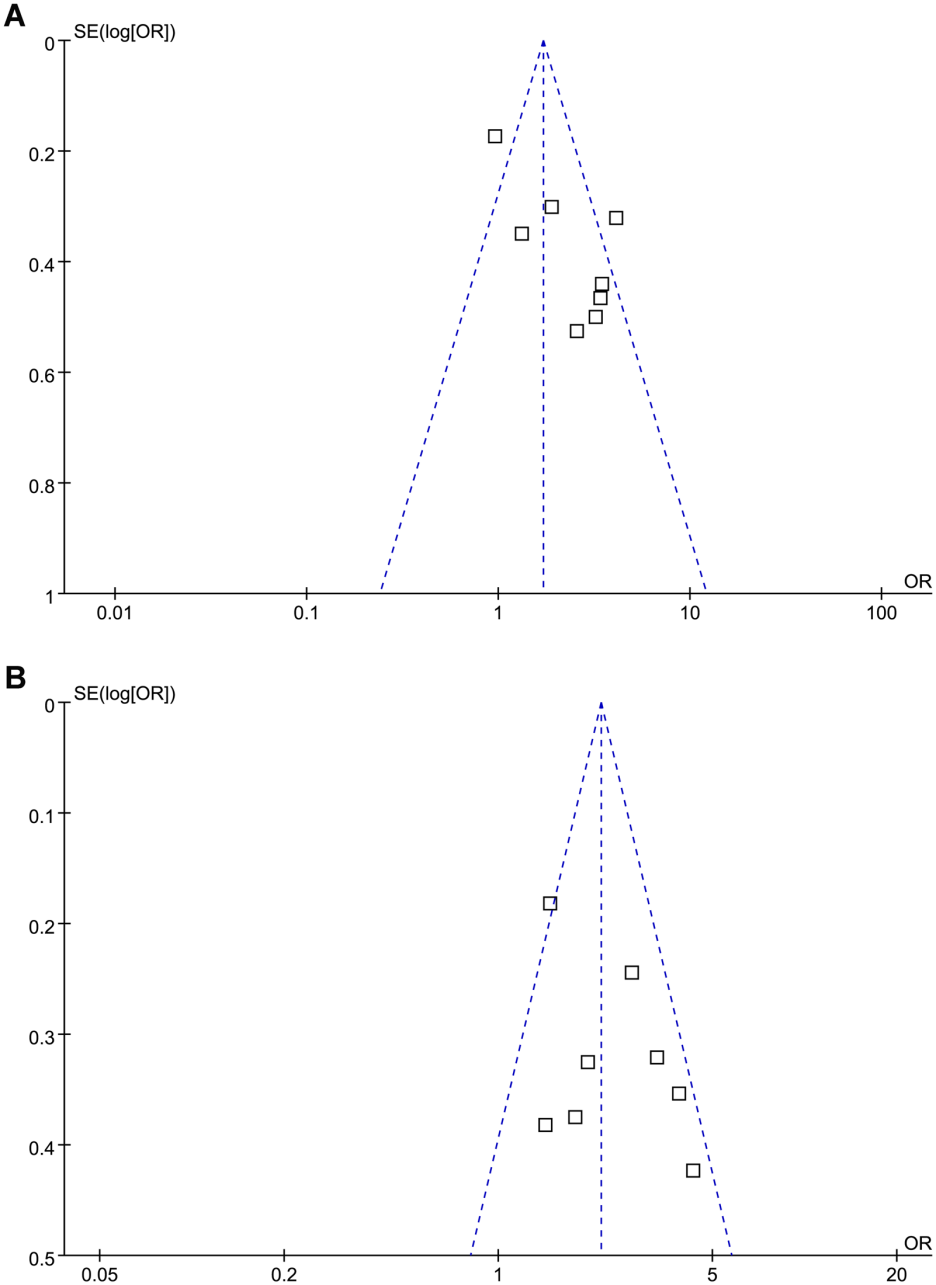
Funnel plots for the meta-analysis of the association between acute GV and mortality risk in patients with acute stroke; **A** funnel plots for the meta-analysis of GV measured by CVBG; and **B** funnel plots for the meta-analysis of GV measured by SDBG;

The funnel plots for the association between CVBG, SDBG and risk of mortality in patients with acute stroke were shown in Fig. 5A and B. On visual inspection, these plots were symmetrical, indicating low risks of publication bias. Egger’s regression test also did not show significant publication biases (p = 0.37 and 0.58, respectively). Publication biases for the meta-analyses with GV measured by MAGE were difficult to estimate because only two studies were included.

## Discussion

In this meta-analysis, we pooled the results of ten cohort studies and the results showed that compared to those with lower acute GV, patients with higher acute GV had a significantly increased risk of mortality within 3 months after the onset of acute stroke. The results were consistent for acute GV measured with CVBG, SDBG, and MAGE. Further subgroup analyses for studies with CVBG and SDBG showed consistent results in patients with AIS and AHS, and in studies evaluating the 28-day and 90-day all-cause mortality. Taken together, the results of the meta-analysis showed that higher acute GV may be an independent risk factor of early mortality in patients with acute stroke. Evaluating acute GV for patients with acute stroke may be important for risk stratification for these patients.

To the best of our knowledge, this is the first meta-analysis that evaluated the relationship between acute GV and subsequent mortality risk in patients with acute stroke. We performed an extensive literature search to obtain relevant studies, and some other strengths of the study should be noticed. Firstly, only cohort studies were included in the meta-analysis, aiming to provide a temporal relationship between higher acute GV and early mortality in these patients. Besides, meta-analyses were performed separately according to the different parameters of acute GV applied, and the consistent results of the meta-analyses further confirmed the robustness of the findings. In addition, only studies with multivariate analyses were included. Accordingly, the results of the meta-analysis showed that the association between higher acute GV and increased risk of early mortality in patients with acute stroke may be independent of the characteristics of the patients, such as the age and NIHSS at baseline. Finally, for studies with acute GV analyzed via CVBG and SDBG, results of subgroup analyses indicated that the association between higher acute GV and increased risk of mortality were consistent for patients with AIS and AHS, and for studies with follow-up durations of 28 days and 90 days. Collectively, these results indicated that acute GV may be an independent predictor of early mortality in patients with acute stroke.

Interestingly, a recent meta-analysis of seven follow-up studies with 725,784 diabetic patients showed that long-term glycemic variability is associated with higher risk of stroke in people with diabetes [37]. Results of our meta-analysis expanded these findings by showing that increased GV is not only involved in the pathogenies of stroke, but is probably also a predictor of poor prognosis in patients with acute stroke. In patients with acute stroke, increased acute GV has been associated with early neurological deterioration [38], poor functional outcome [39], impaired cognitive function [40], higher risk of hemorrhagic transformation [41], and increased incidence of major adverse cardiovascular events [42], all of which may lead to an increased early mortality in these patients. Moreover, evidence from preclinical studies showed that glucose fluctuation is related to pathophysiological changes including oxidative stress, inflammatory response, and endothelial dysfunction etc. [43, 44], all of which are involved in the pathogenesis of recurrent cardiovascular events after stroke. From this point of view, it could be hypothesized that increased acute GV may not be a simple marker of disease severity, but an active participant in the deterioration of stroke. A recent clinical trial showed that targeted intervention to reduce acute glycemic fluctuation was associated with improved nerve function in diabetic patients following AIS [45]. More studies are warranted to determine whether targeted treatment to reduce acute GV could reduce the mortality in patients with acute stroke [46].

## Limitations

Our study also has some limitations. Firstly, all of the included studies were retrospective cohort studies, which may be confounded by selection and recall biases. In addition, limited datasets were available to the meta-analysis of the association between MAGE and mortality in patients with acute stroke. The results of the meta-analysis should be validated in future large-scale prospective studies. Also, the optimal parameter and cutoff of GV evaluation to predict mortality risk in patients with acute stroke remain to be determined, since no consensus has been reached yet in real-world clinical practice. Besides, although only studies with multivariate analyses were included in the meta-analysis, we could not exclude the possibility that there is still residual factor that may confound the association between acute GV and mortality risk, such as the concurrent use of antidiabetic drugs. Finally, a causative relationship between acute GV and mortality risk in patients with acute stroke could not be derived based on our findings as this is a meta-analysis of observational studies. Clinical studies are needed in this regard.

## Conclusions

In conclusion, the results of this meta-analysis showed that higher acute GV may be an independent risk factor of early mortality in patients with acute stroke. Evaluating of acute GV after stroke onset may be important for predicting of prognosis in these patients. Moreover, clinical studies are warranted to determine the possible influence of reducing acute GV on clinical outcomes in patients with acute GV.

## Data Availability

All data generated or analyzed during this study are included in this published article.
